# Enlargements of the Spleen—IV

**Published:** 1908-08-15

**Authors:** 


### Medicine.

### ENLARGEMENTS OF THE SPLEEN—IV.

### TUMOURS THAT MAY BE MISTAKEN FOR AN ENLARGED SPLEEN (*continued*).

*Ovarian Tumours.*—Ovarian tumours have been mistaken for enlargements of the spleen and *vice versa*, but no great difficulty should arise if attention is directed to the following points:—(*a*) An ovarian tumour rarely extends upwards to such an extent that its upper limit comes into actual firm contact with the left costal margin.

(*b*) It does not move downwards to any great extent at the time of a deep inspiration.

(*c*) It has not a sharp well-defined edge with a notch in it, crossing the abdomen obliquely from the left lower ribs downwards and to the right.

(*d*) It extends upwards from the pelvis, the lower part of the abdomen being the more prominent.

(*e*) It alters the position of the umbilicus so that the latter becomes nearer to the ensiform cartilage than to the pubes.

(*f*) If the tumour is very large it occupies both sides of the abdomen almost equally, and it extends much more to the right than an enlarged spleen usually does.

(*g*) On account of its close proximity to the aorta it may receive a transmitted pulsation that may be both seen and felt.

(*h*) A vaginal examination may determine that the cervix and the body of the uterus are pulled upwards.

(*i*) Though dull to percussion in front, it may be found that the dulness is not continuous with that of the ordinary splenic dulness, gastric resonance coming between the two.

Notwithstanding the above points, it is sometimes very difficult to distinguish the two with certainty; thus a woman, aged 18, came under observation for abdominal pains, and for a swelling in the left side of the abdomen, diagnosed as an ovarian cyst. She had noticed the swelling for two years. Latterly there had been increasing anæmia. She did not look ill, however. On abdominal examination a large tumour was found occupying the left side and presenting most of the signs of a splenic enlargement. A blood examination showed 2,500,000 red corpuscles per cub. mm., 80 per cent. of hæmoglobin, and no leucocytosis. A fortnight later there was a severe attack of vomiting and hæmatemesis. The bleeding could not be controlled, and death resulted from the hæmorrhage.

The post-mortem examination revealed a spleen weighing 45 ounces; it was firmly adherent to the under surface of the liver; its substance was resistant and tough, and it contained several infarcts. The liver weighed 37 ounces, and it was a typically hob-nailed cirrhotic organ of the multilobular type. The fatal hæmatemesis was due to rupture of varicose veins at the lower end of the œsophagus. The pelvic organs were macroscopically natural.

*Fæcal Accumulation.*—Fæcal accumulation may be mistaken for an enlargement of the spleen if the splenic flexure of the colon or the adjacent parts of the transverse or descending colon are involved. The points which may help to distinguish it from an enlarged spleen are the following:—(*a*) It is a condition which, in younger persons, is more likely to be found in a woman than in a man; in the aged, men and women are equally affected.

(*b*) There has usually been obstinate constipation and possibly transient attacks of obstruction.

(*c*) Retching or vomiting may be a prominent symptom if the impaction of fæces is marked.

(*d*) The tumour is of an irregular form, generally more or less elongated and cylindrical, but it may vary considerably both in size and shape according to the amount of fæces present. It is important if the shape of the mass varies from day to day, though impacted fæces do not always do so.

(*e*) No definite edge or notch is to be felt.

(*f*) The mass may be movable, and if it is watched its position may be seen to change from time to time.

(*g*) After the administration of purgatives or enemata it may disappear altogether. Its non-disappearance, however, does not always exclude the diagnosis of impacted fæces.

(*h*) On manipulating it with the fingers it may feel like a mass of firm putty, and it may be mouldable into various shapes.

(*i*) On light percussion a dull note may be elicited, but on firmer percussion the note may be resonant.

*Tuberculous Peritonitis.*—In tuberculous peritonitis of the adhesive, fibrous or fibro-caseous forms there may be deceptive lumps in various parts of the abdomen. The transverse puckering of the omentum may form a tumour occupying the left hypochondriac region, simulating an enlarged spleen. The points distinguishing such a mass from an enlarged spleen are: (*a*) The tumour does not as a rule extend close up under the ribs, so that the band may be placed between it and the costal margin.

(*b*) Although it may present a somewhat rounded and well-defined edge, there is seldom a definite notch.

(*c*) The mass may be dull to percussion, but there is likely to be resonance between it and the normal line of splenic dulness.

(*d*) If it is in, or attached to, the abdominal wall there is no downward movement on inspiration.

(*e*) Ascites is frequently present, although, of course, ascites not infrequently accompanies some splenic enlargements also.

(*f*) Masses or tumours may be felt in other parts of the abdomen.

(*g*) Redness of, or even a purulent or fæcal discharge from the umbilicus may be present; tuberculous peritonitis is the commonest cause of spontaneous fæcal fistula at the navel, apart from congenital conditions.

(*h*) Calmette's tuberculo-ophthalmic reaction may be tried, and found positive.

*Malignant Peritonitis.*—All the points just given, except the last, apply also to malignant peritonitis, which occasionally gives a mass in the left hypochondriac region simulating a big spleen. Such peritonitis is nearly always secondary.

